# Standardized freeze-dried FMT: is the ideal protectant out there?

**DOI:** 10.3389/fmicb.2025.1618067

**Published:** 2025-08-13

**Authors:** Alix Pourrat, Vinciane Baillieu, Solene Ansel, Maxime Leonardi, Pierre Poiron, Samuel Bellais, Muriel Paul, Biba Nebbad

**Affiliations:** ^1^Pharmacie à Usage Intérieur, Hôpitaux Universitaires Henri Mondor, Créteil, France; ^2^BIOASTER, Lyon, France

**Keywords:** cryoprotectant, lyoprotectant, trehalose, inulin, lyophilization (freeze-drying), bacterial culture, fecal microbiota transplantation, LIVE/DEAD™

## Abstract

**Background:**

Fecal microbiota transplantation (FMT) is an effective treatment for recurrent *Clostridioides difficile* infections. Freeze-drying offers a next-generation, more practical, and aesthetically acceptable FMT formulation that could facilitate standardized preparation methods. Viable preservation is a critical step in freeze-drying, yet no universal medium effectively protects both anaerobes and aerobes.

**Objective:**

This study aimed to evaluate different protectants compared to trehalose 5% (T5) after confirming its efficacy.

**Methods:**

A mix of inulin and glucosamine (IG5) and a High-antioxidant Matrix with trehalose (HM) were tested. Viability was assessed using colony-forming unit (CFU) enumeration and flow cytometry with a LIVE/DEAD™ staining method.

**Results:**

T5 demonstrated satisfactory bacterial recovery after freeze-drying, with viability of 84 ± 28% for anaerobes and 59 ± 39% for *Bifidobacterium* (BIF), confirming its efficiency in our preparation facilities. While HM showed highest results (91 ± 7% for anaerobes, 121 ± 33% for BIF), it did not significantly outperform T5. IG5, however, resulted in a significant loss of bacteria, with only 16 ± 12% viability for anaerobes (*p* = 0.016) and 19 ± 9% for BIF (*p* = 0.031).

**Conclusion:**

HM and T5 both proved effective for freeze-dried FMT, with HM yielding the highest recovery but not significantly outperforming T5. Given its simplicity and consistent results, T5 may serve as a reliable standalone protectant or as a base for improved formulations. IG5 showed significant bacterial loss and is unsuitable. Further biological validation and stability data will guide the development of optimized freeze-dried oral FMT capsules.

## 1 Introduction

*Clostridioides difficile* infection (CDI) is an urgent antibiotic-associated health threat with limited treatment options. Standard antibiotics such as vancomycin disrupt the gut microbiota, leading to altered microbial community structure, dysbiosis, and a reduction in overall microbial diversity. A new macrocyclic antibiotic, Fidaxomicin, causes less alteration to the bowel microbiota but remain weakly effective on recurrence of infection. Bezlotoxumab was prescribed for the prevention of recurrent CDI (rCDI), but its discontinuation from the market was announced in December 2024. Microbiota restoration through fecal microbiota transplantation (FMT) is an effective treatment option for rCDI (Kelly et al., 2016). While initial FMT methods involved fresh suspensions of stools administered via invasive procedures, advancements have led to the use of frozen suspensions of stools, which offers more convenience and comparable efficacy (Hamilton et al., 2012; Lee et al., 2016).

To date, no fecal transplant currently holds marketing authorization in France; they are considered hospital or magistral preparations. This is not the case in all countries worldwide. In Australia, for instance, the stool bank BiomeBank® obtained marketing authorization in 2022 for its product Biomictra®, a fecal microbiota suspension administered via syringe. Biomictra® is indicated for the restoration of gut microbiota in the management of gastrointestinal disorders. However, its use for the treatment of CDI is limited to the context of registered clinical trials or use under specific regulatory exemptions.

Several stool banks operate internationally, including the prominent U.S.-based OpenBiome®. However, OpenBiome® ceased operations on October 31, 2024, following the U.S. Food and Drug Administration (FDA)’s requirement to submit an Investigational New Drug (IND) application.

In the context of CDI, two fecal microbiota-based products have received FDA approval via the Biologics License Application (BLA) pathway. Fecal Microbiota, Live-jslm (Rebyota®), is administered rectally as a fecal suspension, while Fecal Microbiota Spores, Live-brpk (Vowst®), is delivered orally in the form of four capsules containing purified spores. Rebyota® and Vowst® were approved in 2022 and 2023, respectively, for the prevention—though not the treatment—of rCDI.

Currently, no product on the market holds marketing authorization for the treatment of rCDI outside of clinical trials. Most available formulations are fecal suspensions, except for Vowst®, which is presented in capsule form. Other formulations for FMT have also been developed. The development of frozen stool suspension capsules has enabled oral administration of FMT (Youngster et al., 2014). However, challenges remain in preserving bacterial viability during storage and transport. Moreover, this approach typically requires the ingestion of two doses of 15 large size-00 capsules, which may limit patient compliance. As a result, alternative formulations—such as lyophilized (freeze-dried) stool—are currently being explored to improve stability, ease of administration, and overall acceptability.

Freeze-drying, traditionally the method of choice for producing dry bacterial powders (Miyamoto-Shinohara et al., 2008), represents a promising strategy for FMT formulation. However, the process can compromise the viability of sensitive bacterial species by damaging cell membranes and denaturing proteins. To mitigate these effects, the use of cryo- and lyoprotectants—such as trehalose, mannitol, and skimmed milk—is essential, although their precise protective mechanisms remain incompletely understood. Notably, no universal preservation medium has been identified that effectively stabilizes both anaerobic and aerobic bacteria (Stefanello et al., 2019). In addition, glycerol, a common cryoprotectant for freezing, is unsuitable for freeze-drying due to its high viscosity and poor sublimation properties.

This study aimed to evaluate the protective potential of three formulations to optimize bacterial viability during freeze-drying. An ideal protectant should withstand the key stages of lyophilization (freezing, primary drying by sublimation, and secondary drying by desorption), maintain the viability of key anaerobes, minimize toxicity, be without allergy properties (e.g., skim milk), be cost-effective, and remain easy to apply in microbiome preservation workflows.

Trehalose was tested to confirm its efficacy, as it is widely used as a reference standard. A 5% trehalose concentration has demonstrated effectiveness in clinical studies involving patients with rCDI, with clinical success rates of 43/49 (Staley et al., 2017) and 6/7 patients (Zain et al., 2022). *In vitro*, 5% trehalose maintained a bacterial viable load of 10^8^–10^9^ CFU/g of lyophilized stool, with no statistically significant reduction observed between pre- and post-lyophilization, and no significant viability loss after 36 weeks of storage at −80°C (Zain et al., 2022).

Anhydrous trehalose forms a vitrified matrix with a high glass transition temperature (~100°C), stabilizing cell membranes and proteins by forming hydrogen bonds and reducing ice crystal formation (Crowe et al., 1996; Herdeiro et al., 2006). Despite these properties, trehalose is not universally effective. Its protective efficacy varies by concentration and strain, with some bacterial taxa still vulnerable to intracellular damage during freeze-drying. For instance, Jofré et al. reported that 5% trehalose alone caused moderate viability loss in *Lactobacillus rhamnosus* CTC1679. The addition of 11% skim milk significantly enhanced preservation, reducing viability loss to nearly baseline levels (Jofré et al., 2015). Similarly, Wang et al. showed that soy polysaccharide improved the survival of *Lactiplantibacillus plantarum* AR113 by 19% compared to trehalose alone. A composite of soy polysaccharide and trehalose achieved a 90.5% survival rate for *L. plantarum* WCFS1—an increase of over 30% relative to either agent alone—likely due to better preservation of membrane integrity and enzyme activity (Wang et al., 2021). These findings underscore the limitations of single-component cryoprotectants or lyoprotectants and the potential advantages of synergistic formulations combining micromolecular and macromolecular agents. In a similar approach, Bellali et al. developed a complex protectant medium composed of sucrose, skimmed milk, trehalose, CaCl_2_, MgCl_2_, KOH, and three antioxidants (ascorbic acid, uric acid, and glutathione). Their formulation significantly enhanced the preservation of total anaerobic bacteria compared to unprotected samples, which they attributed to antioxidant-mediated protection against oxygen exposure. Notably, they reported improved recovery of fastidious and extremely oxygen-sensitive (EOS) taxa such as *Treponema denticola*, *Methanobrevibacter smithii*, and *Faecalibacterium prausnitzii* (data unpublished) (Bellali et al., 2020).

While trehalose offers effective protection, studies have shown that higher concentrations (e.g., up to 15%) can markedly improve survival rates of lactic acid bacteria and yeast during freeze-drying and storage (Rockinger et al., 2021; Stefanello et al., 2018). However, increasing trehalose content may not always be feasible or sufficient.

This highlights the need for complementary agents that enhance protection without relying solely on high trehalose doses.

In light of this, we explored whether trehalose-based protection could be enhanced by incorporating additional functional components.

In this end, we tested a locally developed, antioxidant-enriched formulation (High-antioxidant Matrix: HM) that combines trehalose with ascorbic acid, sorbitol, and glutamate to mitigate oxidative stress.

In parallel, we formulated IG5, a novel composite of 5% inulin and 5% glucosamine.

The combination of a long-chain polysaccharide, such as maltodextrin, with a short sugar like sucrose or trehalose, has been shown to enhance stability during lyophilization, due to the high glass transition (Tg) of the long-chain polysaccharide (Bai, 2024). This high Tg may help stabilize cellular membranes and limit ice crystal formation (Rodríguez Furlán et al., 2011). Hence, a freeze-dried stool formulation containing 6.7% maltodextrin and 10% trehalose demonstrated the best post-lyophilization viability based on CFU counts for multiple taxa (*Enterococci*, *Enterobacteria*, *Bacteroides*, *Bifidobacteria*, and *Clostridioides*) compared to 10% trehalose alone (Kapel et al., 2017). Moreover, a maltodextrin–trehalose combination (patent WO2017103225A1) proved effective both *in vitro* and *in vivo* against *C. difficile* (Reygner et al., 2020).

Inulin is also a long-chain polysaccharide with a high Tg. Similarly, association of inulin and sucrose significantly improved post-lyophilization viability in six isolated strict anaerobic gut bacteria (*Faecalibacterium prausnitzii*, *Roseburia intestinalis*, *Anaerostipes caccae*, *Bacteroides thetaiotaomicron*, *Eubacterium halii*, *Blautia obeum*) compared to samples without any protectant (Bircher et al., 2018). Inulin’s protective effect was mainly observed during lyophilization, with limited efficacy during long-term frozen storage (Bircher et al., 2018; Oluwatosin et al., 2021). Inulin is a prebiotic soluble fiber that promotes the growth of beneficial gut microorganisms (Qin et al., 2023), particularly *Bifidobacterium* and *Lactobacillus*, contributing to gut health by enhancing microbiome diversity. Not digested in the small intestine, inulin reaches the colon intact, where it is fermented by gut bacteria, producing short-chain fatty acids (SCFAs) that offer numerous health benefits (Tawfick et al., 2022). As previously mentioned, in addition to its prebiotic role, inulin has shown potential lyoprotective properties under certain conditions (Oluwatosin et al., 2021). However, its effectiveness as a cryoprotectant remains poorly documented (Hubálek, 2003), and may be limited by its relatively high molecular weight and complex polymeric structure compared to more conventional protectants.

In association to inulin, in this study, we explored an alternative to trehalose—an expensive sugar—by investigating the use of glucosamine, an amino sugar, as the short sugar component for membrane protection, and given its recognized antioxidant properties (Fernández-Rojas et al., 2023; Xing et al., 2006). Although glucosamine is not a acknowledged cryoprotectant, its sugar-like structure suggests it may confer partial protection during freeze-drying by stabilizing proteins. Like other sugars, amino sugars can form hydrogen bonds with biological macromolecules, replacing water molecules and potentially stabilizing cellular structures during dehydration (Hubálek, 2003). In addition, glucosamine is a structural component of bacterial cell walls and contributes to their integrity through its involvement in peptidoglycan and lipopolysaccharide biosynthesis.

Although initial tests showed limited efficacy when inulin and glucosamine were used individually (data not shown), their combination in IG5 was hypothesized to provide synergistic benefits.

Unlike conventional agents such as skim milk or sucrose, which provide broad but nonspecific protection, HM and IG5 were specifically formulated to offer targeted protection. Their antioxidant and prebiotic properties may synergistically preserve oxygen-sensitive taxa during lyophilization.

The objective was to develop a practical and effective protectant for anaerobic bacteria essential to FMT in rCDI (Baktash et al., 2018; van Nood et al., 2013).

## 2 Materials and methods

This study was conducted at the Henri Mondor Fecal Microbiota Preparation Unit to evaluate bacterial viability before and after freeze-drying, with the aim of assessing the protective efficacy of three protectant formulations (Threalose 5% (T5), HM, and IG5). Viability was assessed using two complementary methods: colony-forming unit (CFU) enumeration under both aerobic and anaerobic conditions, and flow cytometry combined with LIVE/DEAD™ staining to differentiate live and dead bacteria. Detailed protocols for each method are described below.

### 2.1 Collection of stool samples

Fecal samples were collected from two healthy volunteers. Donors were screened based on self-reported health history, including absence of gastrointestinal disorders, a normal body mass index, and no antibiotic use within the past 6 months. No additional regulatory donor screening procedures were performed, such as infectious disease testing or screening for asymptomatic pathogen carriage. Samples were collected in clean containers, and, if transport was required, an anaerobic generator (AnaeroGen Compact®; Thermo Scientific) was used. Samples were either processed within 2 h of collection or immediately frozen at −80°C to preserve microbial viability.

### 2.2 Preparation of stools for the freeze-drying procedure

Fecal samples were prepared, following Hamilton’s procedure (Hamilton et al., 2012). All preparation steps were carried out under aerobic conditions but the material used for the suspension of stools as well as its homogenization allowed us to expose the raw material to air as little as possible (closed dilution system, homogenization of the stool in the closed BagFilter®). Stools preparations were performed in a dedicated facility, under a clean hood. Stools were weighed in an irradiated BagFilter® (Interscience, Saint-Nom-la-Bretèche, France) ranging from 21 g to 78 g (median 44.5) then diluted at a 1:4 (v/v) ratio with cold saline stored at +4°C, using the gravimetric dilutor DiluFlow® (Interscience). The samples were then homogenized automatically with the BagMixer® device (BagMixer 400S, Interscience), for 6 min. The lateral filter integrated to BagFilter® (280 μm) allowed for direct filtration of the stool during homogenization, removing cellular debris and undigested food fibers. The filtrate was centrifuged at 5,000 rpm for 20 min at +5°C. The resulting pellets were resuspended in 1:1 (v/v) in each protectant solution. These suspensions were placed in a lyophilizer (Lyophiliser alpha 2–4 lscplus-102142-christ-Germany). Part of the suspension was used for microbiological culture, while another part was used for cytometry analysis in the T5-treated samples.

### 2.3 Protectants composition

Three protectants formulation were tested:

- T5: Trehalose 5% (Sigma-Aldrich 6138-23-4) in Phosphate Buffered Saline (PBS)
- IG5: Inulin 5% (vit4ever®, Stolberg, Germany) + glucosamine 5% (vit4ever®, Stolberg, Germany) in PBS
- HM: Trehalose 8% + ascorbic acid 0.5% (Laroscorbine®, Bayer HealthCare SAS, Germany) + sorbitol 5% (Sorbitol Delalande®, Sanofi, France) + glutamines 0.5% (Farmalabor, Italy) in Sterile Water (Versol®, Aguettant, France)

T5 and IG5 formulations were tested using samples from both donors, whereas HM was evaluated using samples from only one donor due to limited availability. All protectant conditions were processed and analyzed using identical protocols. Where applicable, results represent combined data from both donors; HM results correspond exclusively to a single-donor stools samples.

### 2.4 Freeze-drying process

The freeze-drying process involved a sequential series of steps: freezing at −80°C for 5 h (1,013 mbar), primary desiccation at −20°C for 16 h (0.133 mbar), and secondary desiccation at +30°C for 3 h (0.133 mbar).

### 2.5 Viability evaluation

#### 2.5.1 Enumeration of fecal material using the petri dish method

Bacterial counts were performed using the spiral plating technique with the EasySpiral Dilute® system (Interscience), a method standardized under ISO 7218 (International Organization for Standardization, 2024). Enumeration was conducted on both fresh stool suspensions containing the protectant formulations and freeze-dried suspensions reconstituted in PBS. The EasySpiral Dilute® system automatically performs serial 1:10 dilutions up to 10^−5^. From the final dilution, the system carries out spiral plating by depositing a logarithmically decreasing volume of sample onto a rotating Petri dish, enabling four logs of dilution to be represented on a single plate. Each plating was performed in duplicate to ensure repeatability. A Tryptone Soy Agar (TSA) plate inoculated with sterile saline was used as a negative control. Five types of culture media were used. Following spiral plating after serial dilutions (up to 10^−5^ for most bacteria and up to 10^−3^ for *Lactobacillus*), plates were incubated under aerobic or anaerobic conditions using an anaerobic generator (AnaeroGen Compact®, Thermo Fisher Scientific). All media were incubated at 37°C.

Culture Media and Incubation Conditions:

- TSA (Becton Dickinson, Franklin Lakes, New Jersey, USA): were used for total microbiota enumeration. Plates were incubated aerobically for 24 h.
- Drigalski agar (DRI, bioMérieux, France): were used for selective isolation of coliforms, incubated 24 h in aerobic condition.
- Columbia Horse Blood agar (COH; bioMérieux; France): were used for total anaerobes bacteria. Plates were incubated anaerobically for 48 h.
- *Bifidobacterium* agar (BIF, Becton Dickinson, USA): were used for selective cultivation of *Bifidobacterium* spp. Plates were incubated anaerobically for 48 h.
- *Lactobacillus* Selective agar (LBS, Becton Dickinson, USA): were used for the selective isolation of *Lactobacillus* spp. Plates were incubated anaerobically for 48 h.

Due to product discontinuation, COH agar was replaced with Columbia Sheep Blood Agar (COS; Thermo Fisher Scientific, USA) after three complete experiments with the T5 formulation and two with the HM formulation (Table 1).

**Table 1:** Number of samples analyzed for every protectant formulation with six different media agar.

Protectant	TSA	DRI	COH	COS	BIF	LBS
Trehalose 5%	8	8	3	5	5	2
HM	4	4	2	2	4	1
IG5	7	7	0	7	6	3

T5, Trehalose 5%; HM, High-antioxidant Matrix, Trehalose- ascorbic acid-sorbitol-glutamine; IG5, Inulin-Glucosamine TSA, Tryptone Soy Agar for total aerobe bacteria; COH/COS, Columbia Blood Horse/Sheep agar for anaerobes; DRI, Drigalski for Enterobacteria; BIF, *Bifidobacterium* spp.; LBS, *Lactobacillus* spp.

**Figure 1:**
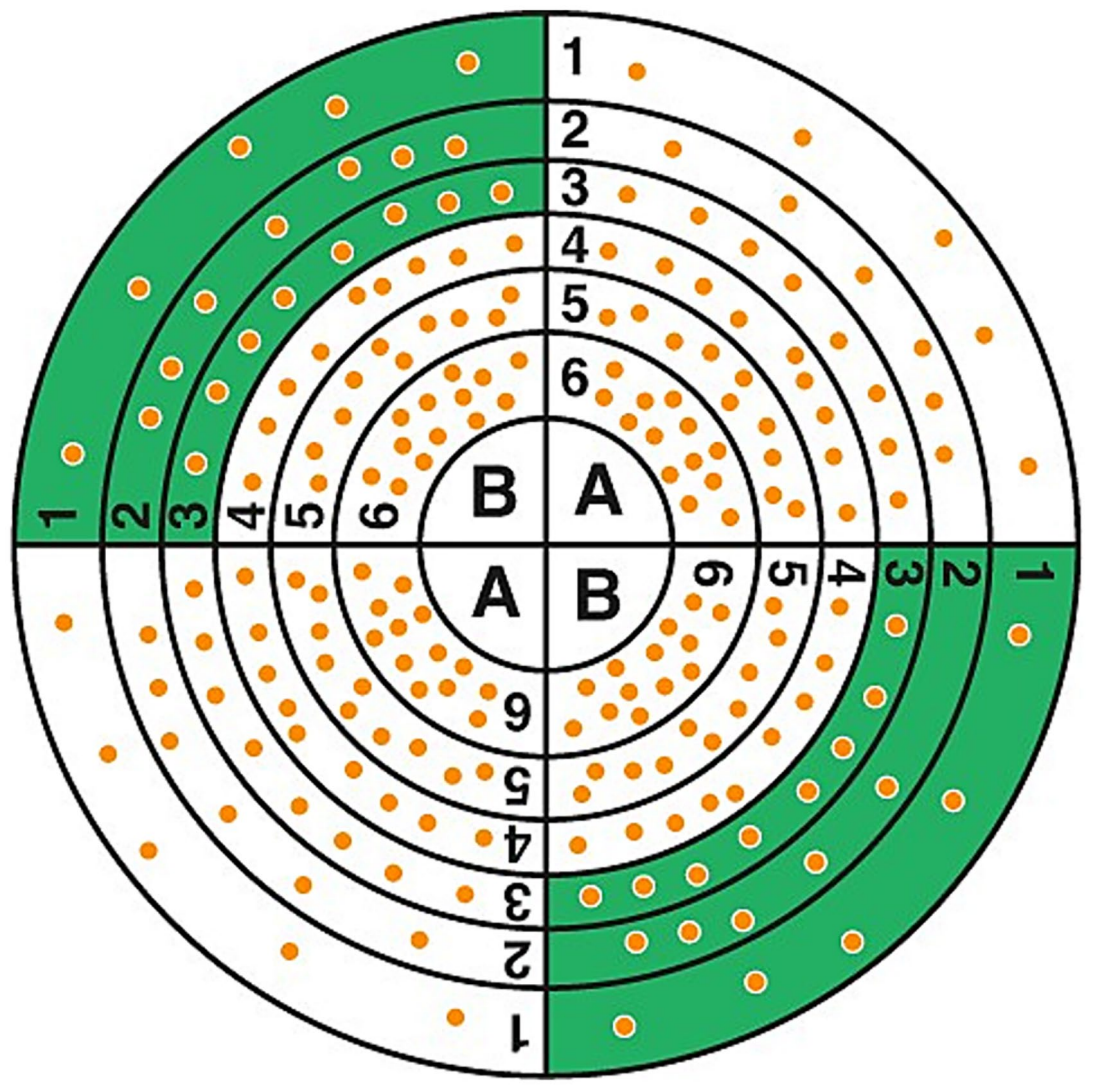
Bacterial counting grid. At least 20 colonies should be counted in the outer sectors, with the process repeated on the opposite side. The bacterial concentration was determined by dividing the colony count by the deposited volume.

Counting was carried out manually using a grid by two independent operators to ensure double control (Figure 1).

Viability was calculated as the percentage ratio of viable cells after and before freeze-drying, based on the total CFU count measured in the entire lyophilized powder and in the whole pellet. It was calculated using the following equation (Huang et al., 2006)


$$\text{Viability}\;(\%)=\frac{\text{Live cells after freeze}-\text{drying}\;(\mathrm{CFU})}{\text{Live cells before freeze}-\text{drying}\;(\mathrm{CFU})}\times 100$$

#### 2.5.2 Enumeration of fecal samples by flow cytometry

Due to its high cost, flow cytometry was only performed on samples added with T5.

For six T5-added samples, bacterial viability was assessed using both CFU enumeration and flow cytometry with the LIVE/DEAD™ BacLight™ Bacterial Viability Kit (SYTO™ 9/Propidium Iodide (PI); Thermo Fisher Scientific), following the manufacturer’s instructions. A freshly collected stool sample from an unrelated donor was included as a control.

After staining, samples were washed with PBS and analyzed within 30 min using an Influx® cell sorter (Becton Dickinson) equipped with 488 nm, 640 nm, and 405 nm lasers. Three bacterial subpopulations were identified based on membrane integrity:

- Intact-membrane cells (considered viable): SYTO™ 9-stained
- Membrane-permeable cells (considered non-viable): PI-stained
- Intermediate cells (partially damaged): double-stained with SYTO™ 9 and PI

The analysis was limited to two pairs of samples (one before and one after freeze-drying) and two additional independent freeze-dried samples.

All samples were analyzed in duplicate.

### 2.6 Statistical analysis

Comparison of total CFU counts before and after freeze-drying with a given protectant was performed using a paired permutation test, a test well-suited for small sample sizes and non-normally distributed data (Nichols and Holmes, 2007). Comparisons of viability between T5 and HM, and between T5 and IG5, were also carried out using a permutation test. Differences were considered statistically significant when the *p*-value was less than 0.05.

For flow cytometry data, comparisons between CFU/g and MO/g (measured in both grams of lyophilized and grams of pre-lyophilized stool samples) were performed using simple linear regression analysis. Due to the limited number of measurements (five data points, each with a single replicate), this analysis was conducted with the aim of evaluating the existence of a potential linear relationship, rather than establishing statistical significance. CFU/g values represent the combined counts of total aerobes (TSA) and total anaerobes (COH or COS).

## 3 Results

### 3.1 Microbiota viability in freeze-dried and fresh fecal samples with trehalose 5%

#### 3.1.1 Enumeration of samples by plate method

**Figure 2:**
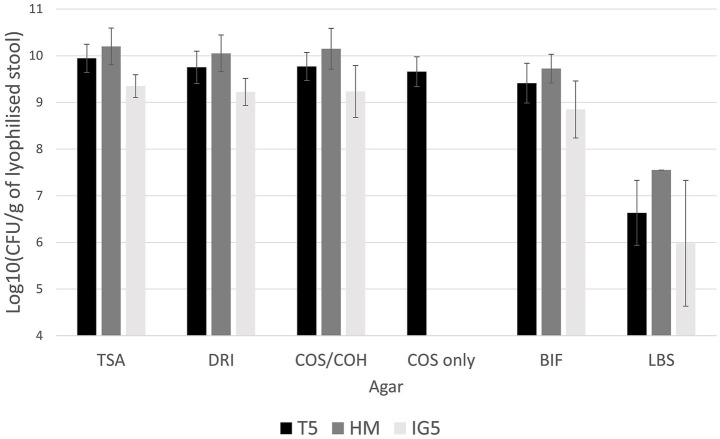
Mean bacterial counts after lyophilization on T5 (trehalose); HM (trehalose-sorbitol-glutamine) and IG5 (inulin-glutamine) protectant (Colony-forming Unit CFU/g of lyophilized stool). No statistical analysis was conducted, as each protectant was tested on a different stool sample, making direct comparisons inappropriate. TSA, Tryptone Soy Agar for total aerobe bacteria; COH/COS, Columbia Blood Horse/Sheep agar for anaerobes; DRI, Drigalski for Enterobacteria; BIF, *Bifidobacterium* spp.; LBS, *Lactobacillus* spp.

The resulting counts of total aerobes, total anaerobes, and coliforms ranged from 10^9^ to 10^11^ CFU per gram of lyophilized stool across all protectants. *Bifidobacterium* spp. were slightly less abundant, ranging from 10^8^ to 10^10^ CFU/g. while *Lactobacillus* spp. were present at lower levels between 10^6^ and 10^8^ CFU/g (Figure 2).

As shown in Table 2, freeze-drying did not significantly impact bacterial viability when using the T5 protectant. However, slight differences were observed depending on the culture medium.

**Table 2:** Bacterial viability by plating (Colony Forming Unit CFU) before freeze-drying in the total stool pellet and after freeze-drying in the total lyophilized stool obtained with the T5 (trehalose) protectant.

Medium	Bacterial count before freeze-dry in pellet (CFU)	Bacterial count after freeze-dry in lyophilized stool (CFU)	Number of samples	Viability (%)	*p*-value
TSA	(3.59 ± 2.94) ×10^10^	(4.40 ± 4.22) ×10^10^	8	118 ± 35	0.109
DRI	(2.71 ± 1.96) ×10^10^	(2.93 ± 2.61) ×10^10^	8	108 ± 50	0.594
COH/COS	(3.57 ± 2.210) ×10^10^	(2.96 ± 2.60) ×10^10^	8	84 ± 28	0.141
COS only	(3.23 ± 1.80) ×10^10^	(2.09 ± 1.56) ×10^10^	5	69 ± 24	0.125
BIF	(2.35 ± 1.48) ×10^10^	(1.83 ± 1.78) ×10^10^	5	59 ± 39	0.188
LBS	(3.83 ± 4.65) ×10^7^	(3.47 ± 4.41) ×10^7^	2	78 ± 21	0.500

Statistical difference between before and after was assessed by paired-permutation tests.

TSA, Tryptone Soy Agar for total aerobe bacteria; COH/COS, Columbia Blood Horse/Sheep agar for anaerobes; DRI, Drigalski for Enterobacteria; BIF, *Bifidobacterium* spp.; LBS, *Lactobacillus* spp.

T5 maintained high viability on TSA (118 ± 35%) and Drigalski (108 ± 50%) agars. For bacteria of particular interest in the treatment of rCDI—primarily anaerobes cultured on COH or COS agar—viability was slightly lower but still acceptable at 84 ± 28%, with no statistically significant decrease (*p* = 0.141). This result remained non-significant even when the three COH samples were excluded (*p* = 0.125). A satisfactory viability was also observed for *Bifidobacterium* spp. (59 ± 39%). Regarding *Lactobacillus* spp., only two data points were considered reliable (78 ± 21%), as counts were either too high to isolate individual colonies or too low for accurate enumeration.

HM showed no significant decrease of bacteria after lyophilization for all media tested. For anaerobes, we obtained a similar mean bacterial count before and after freeze-drying (1.59E+11 *versus* 1.41E+11, *p* = 0.250) (Table 3).

**Table 3:** Bacterial viability by plating (Colony Forming Unit CFU) before freeze-drying in the total stool pellet and after freeze-drying in the total lyophilized stool obtained with the HM (High-antioxidant Matrix: Trehalose- ascorbic acid-sorbitol-glutamine) protectant.

Medium	Bacterial count before freeze-dry in pellet (CFU)	Bacterial count after freeze-dry in lyophilized stool (CFU)	Number of samples	Viability (%)	*p*-value
TSA	(1.61 ± 2.67) ×10^11 a^	(1.42 ± 1.98) ×10^11 a^	4	151 ± 76	0.875
DRI	(1.16 ± 1.79) ×10^11 a^	(1.05 ± 1.48) ×10^11 a^	4	117 ± 55	0.750
COH/COS	(1.59 ± 2.37) ×10^11^	(1.41 ± 2.09) ×10^11^	4	91 ± 7	0.250
BIF	(2.82 ± 2.14) ×10^10^	(2.97 ± 1.60) ×10^10^	4	121 ± 33	0.625

Statistical difference between before and after was assessed by paired-permutation tests.

TSA, Tryptone Soy Agar for total aerobe bacteria; COH/COS, Columbia Blood Horse/Sheep agar for anaerobes; DRI, Drigalski for Enterobacteria; BIF, *Bifidobacterium* spp.; LBS, *Lactobacillus* spp.

^a^Although the mean values appear higher before freeze-drying than after, viability and *p*-value were calculated using paired samples, see Supplementary material and Table 2.

Lastly, viability was significatively decreased in all media tested (*p*-value <0.05) for IG5 (Table 4).

**Table 4:** Bacterial viability by plating (Colony Forming Unit CFU) before freeze-drying in the total stool pellet and after freeze-drying in the total lyophilized stool obtained with the IG5 (inulin-glucosamine) protectant.

Medium	Bacterial count before freeze-dry in pellet (CFU)	Bacterial concentrations after freeze-dry in lyophilized stool (CFU)	Number of samples	Viability (%)	*p*-value
TSA	(4.15 ± 3.28) ×10^10^	(1.41 ± 8.25) ×10^10^	7	48 ± 28	0.016*
DRI	(3.11 ± 1.85) ×10^10^	(1.13 ± 7.56) ×10^10^	7	42 ± 21	0.016*
COH/COS	(1.34 ± 1.05) ×10^11^	(1.77 ± 1.60) ×10^10^	7	16 ± 12	0.016*
BIF	(4.34 ± 5.46) ×10^10^	(8.02 ± 7.38) ×10^9^	6	19 ± 9	0.031*
LBS	(4.61 ± 7.77) ×10^8^	(2.18 ± 3.16) ×10^8^	3	11 ± 4	0.250

Statistical difference between before and after was assessed by paired-permutation tests (*p-value < 0.05).

TSA, Tryptone Soy Agar for total aerobe bacteria; COH/COS, Columbia Blood Horse/Sheep agar for anaerobes; DRI, Drigalski for Enterobacteria; BIF, *Bifidobacterium* spp.; LBS, *Lactobacillus* spp.

#### 3.1.2 Enumeration of T5 samples by flow cytometry coupled with the LIVE/DEAD™ method

**Figure 3:**
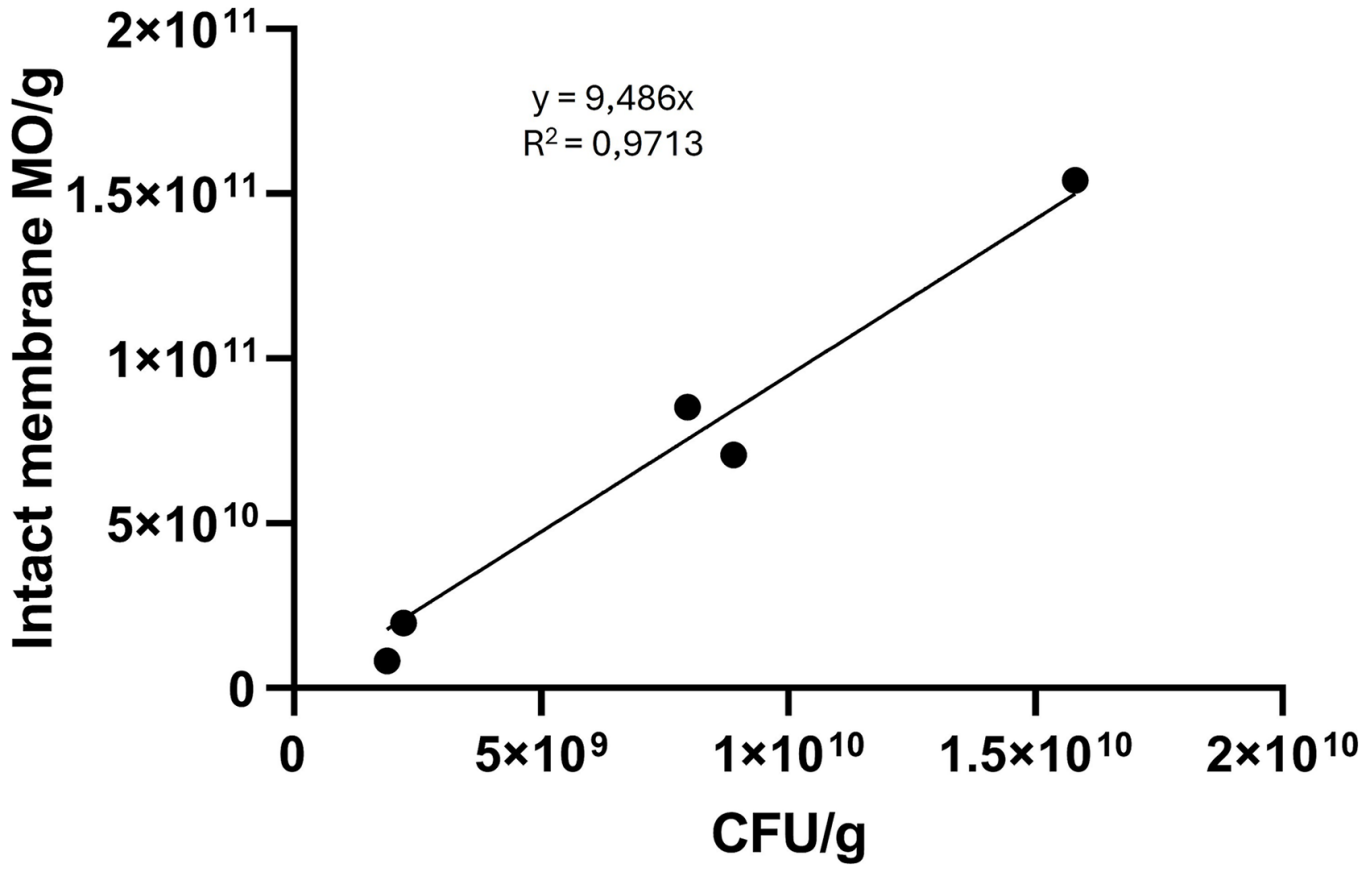
Linear regression between CFU/g quantified by plating and microorganisms (MO)/g quantified by flow-cytometry. When the sample is lyophilized, the weight in grams refers to grams of lyophilized stool. When the sample is a pellet, the weight refers to the pre-lyophilized material (pellet + protectant).

Microorganism counts per gram measured by flow cytometry and CFU/g values obtained by plating appeared to correlate linearly (*y* = 9.486x; *R*^2^ = 0.9713; F-test = 368.8; *p* < 0.0001) (Figure 3). The assumptions for linear regression were met: the relationship was approximately linear by visual inspection; residuals were independent and normally distributed (as confirmed by the Shapiro–Wilk test); and homoscedasticity was confirmed. One sample, analyzed only after freeze-drying, was excluded from the regression due to aberrant values (sample 4, Supplementary Table 1).

**Figure 4:**
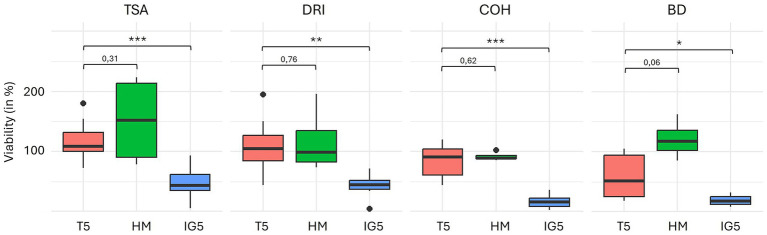
Viability percentage between T5 (trehalose) and HM (trehalose-sorbitol-glutamine), and between T5 and IG5 (inulin-glutamine) protectant. Permutation test were used (*p-value < 0.05; **p-value < 0.01; ***p-value < 0.0001). TSA, Tryptone Soy Agar for total aerobe bacteria; COH/COS, Columbia Blood Horse/Sheep agar for anaerobes; DRI, Drigalski for Enterobacteria; BIF, *Bifidobacterium* spp.; LBS, Lactobacillus spp.

The total microorganism count per gram of lyophilized or pre-lyophilized stool ranged from 10^10^ to 10^12^. Overall viability in lyophilized stool, assessed by membrane integrity, was 21.1 ± 5.6% (Figure 4; Supplementary Table 1).

An average viability ratio of 79% was observed between pellet and lyophilized forms in paired samples. Although the fresh stool samples were not from the same donors as the lyophilized samples, data suggest that the greatest loss of viability occurs during the initial stool processing steps—up to the first centrifugation—evidenced by a preservation rate of only 41% (Table 5).

**Table 5:** Percentage of live bacteria obtained with flow cytometry coupled with the LIVE/DEAD™ method.

Percentage of live bacteria				Preservation (P/FS)	Preservation (L/P)
Samples	Fresh Stool (FS)	Pellet (P)	Lyophilizate (L)		
Sample 1	75%	23%	19%	31%	80%
Sample 2		37%	29%	50%	78%
Mean		30%	24%	41%	79%

See enumeration numbers on Supplementary Table 1.

### 3.2 Comparison of protectants

Although viability seemed higher with the HM protectant, no statistically significant difference was found between HM and T5.

**Figure 5:**
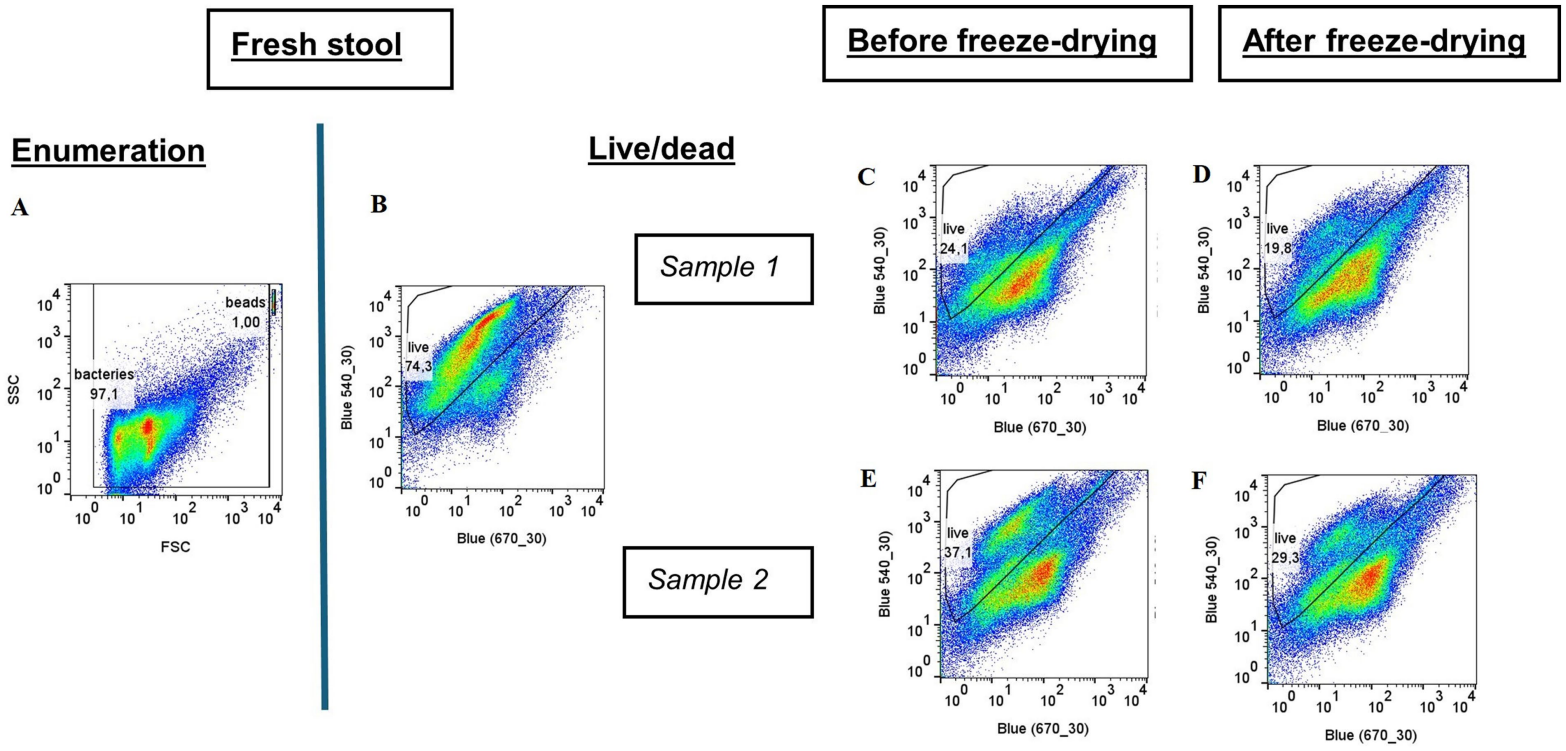
Flow cytometry results for fresh stool and for both paired samples before and after freeze-drying. **(A)** Total flow cytometry of fresh stools; Flow cytometry coupled with the live/dead method: **(B)** Fresh stool; **(C,D)** sample 1 before and after freeze-drying; **(E,F)** sample 2 before and after freeze-drying.

In contrast, IG5 was less effective than T5 in preserving viability, showing highly significant decreases for total aerobes and total anaerobes (*p* < 0.001), as well as significant reductions for coliforms (*p* = 0.004) and *Bifidobacterium* (*p* = 0.048) (Figure 5). Viability of *Lactobacillus* remained similar between groups (n_T5_ = 2 and n_IG5_ = 3; *p* = 0.2).

## 4 Discussion

T5 and HM protectants tested in this study showed satisfactory results in preserving bacterial viability during freeze-drying. However, IG5—the only formulation without established cryoprotective properties in the literature—demonstrated poor performance across all bacterial groups examined.

T5 consistently preserved viability across Enterobacteria, strict anaerobes, and *Bifidobacterium*, suggesting broad-spectrum protection. This effect is likely due to trehalose’s dual function: intracellular stabilization of membranes and proteins, and formation of an extracellular vitrified matrix that inhibits ice crystal formation. Our results align with those of Staley et al., who found 5% trehalose to be clinical effective in rCDI (Staley et al., 2017). However, their study relied solely on membrane integrity assays using microscopy on a single sample, whereas we employed both culture-based enumeration and flow cytometry, providing a more comprehensive assessment. Even though trehalose alone showed no statistically significant difference before and after lyophilization for anaerobes, the boxplots revealed a trend toward 60–100% viability. This viability appeared higher with HM, although the difference was not statistically significant.

HM was designed to enhance anaerobic protection through mitigation of oxidative stress. It demonstrated the highest overall viability across all tested taxa, including both anaerobes and more oxygen-tolerant bacteria, suggesting a synergistic effect.

Comparable results were reported by Bellali et al., who also developed a complex protectant medium containing three antioxidants (ascorbic acid, uric acid, glutathione), among other components (Bellali et al., 2020).

Our results did not demonstrate a clear advantage of HM over T5. However, for *Bifidobacterium*—a genus known for its oxygen sensitivity—the comparison between HM and T5 yielded a *p*-value of 0.06 and a boxplot trend that could suggest a better viability with HM. Although this difference was not statistically significant, it may could reach significance with a larger sample size. While the compositions differ, both studies nonetheless highlight the possible role of antioxidant supplementation in improving the post-lyophilization viability of EOS bacteria.

A potential confounding factor exists: the HM formulation differed from T5 not only in antioxidant content but also in trehalose concentration (8% vs. 5%). Staley et al. reported comparable membrane integrity–based viability between 5 and 10% trehalose, as assessed by microscopy on single samples (Staley et al., 2017). Similarly, no significant difference in CFU counts was observed across three replicates for both concentrations (Kapel et al., 2017). Although our 5% trehalose condition showed no viability loss before and after freeze-drying, the absence of a direct comparison with 8% trehalose alone prevents us from determining whether the improved bacterial viability stems from antioxidant supplementation, increased trehalose concentration, or a combination of both.

Ultimately, in this formulation, HM’s greater complexity and lack of clear superiority over T5, limit its immediate practical value.

For IG5, although the combination of inulin—a longer-chain polysaccharide—with a shorter sugar such as glucosamine might be expected to enhance stability due to its high Tg (Bai, 2024), this was not observed in our study. An antagonistic interaction between glucosamine and inulin could be hypothesized. Nevertheless, inulin alone (data not shown) did not result in high post-lyophilization viability. It is also possible that inulin’s protective effect is more effective when the formulation is incubated in culture prior to lyophilization [24 h hours of incubation in culture medium for a *Lactobacillus plantarum* probiotics (Oluwatosin et al., 2021) and 30 min for six anaerobic gut bacteria (Bircher et al., 2018)]. Indeed, high molecular weight of inulin may require a certain exposure time to interact effectively with cells before processing. However, such protocols do not reflect current practices in fecal transplant preparation, although they could be further explored. IG5 which lacks a structural matrix-forming component such as trehalose that can create a protective glassy matrix during drying, likely underperformed due to insufficient extracellular and intracellular protection throughout the lyophilization process. This discrepancy may stem from glucosamine’s weaker and more variable antioxidant capacity (Fernández-Rojas et al., 2023). These limitations may explain IG5’s reduced efficacy across bacterial groups. For future investigation, trehalose—a well-established cryoprotectant—may constitute a more suitable partner for inulin or another less expensive short-chain sugar as sucrose. Their combined protective effect during lyophilization should also be assessed for long-term storage stability at different temperatures.

Flow cytometry results matched CFU counts, confirming the reliability of our approach. Although no statistical analysis was done with the two paired-samples, no substantial loss of viability was observed between the bacterial pellet (sampled just before lyophilization) and the final freeze-dried product. This suggest that freeze-drying itself is not the main source of bacterial death. Indeed, the greatest loss of viability occurred between fresh stool and the pre-lyophilized pellet, underscoring upstream sample preparation as the critical bottleneck. While fresh stools sample showed an average viability of 75%, an estimated 41% loss occurred by the time pellets were prepared. Though the fresh stool did not correspond to the exact same sample as the pellet, this estimate aligns with literature reporting 40–80% viability in fresh fecal samples (Bilinski et al., 2020). Improving pre-freeze-drying handling may thus have a greater impact than optimizing cryo- and lyoprotectants alone.

Sample size varied across formulations. HM was evaluated in only four samples and assessed solely through culture-based methods. IG5 was similarly analyzed using only CFU enumeration. In contrast, eight T5-treated samples were tested, two pairs of which underwent both CFU counting and flow cytometry using the LIVE/DEAD™ BacLight™ Bacterial Viability Kit. This staining method is based on membrane permeability, yet several limitations have been noted (Berney et al., 2007). For instance, dormant cells may appear PI-positive despite being viable, depending on membrane state and permeability. Conversely, some cells in exponential growth can transiently take up PI, leading to an overestimation the dead fraction (Cabeen and Jacobs-Wagner, 2005; Sträuber and Müller, 2010). Additionally, SYTO™9 is not always fully displaced by PI, which can result in an overestimation of live cells. Furthermore, its penetration is known to be less efficient in Gram-negative bacteria (Stiefel et al., 2015). Despite these caveats, the concordance observed between culture-based and flow cytometry-based measurements supports the reliability of culture-derived viability estimates for HM and IG5. The observed ~1 log difference in microbial counts and the ~12% discrepancy in viability percentages (91% by CFU vs. 79% by flow cytometry) are expected, considering that flow cytometry SYTO™9/PI staining detect also non-bacterial cells such as archaea (Heise et al., 2016) and fungi (Zhang and Fang, 2004). Moreover, it is well known that 60–80% of bacteria are non-cultivable using standard culture methods (Eckburg et al., 2005; Suau et al., 1999). Nonetheless, the absence of parallel flow cytometric data for HM and IG5 remains a methodological limitation that may affect the comparability of viability outcomes across formulations. Moreover, a greater number of samples and replicates is needed to perform a complete regression analysis assessing the concordance between plating and flow cytometry.

A technical variable was introduced by a shift in culture media from COH to COS due to commercial availability. Three T5 and two HM samples were prepared using COH medium. The three T5 samples exhibited the highest viability values, which may be attributable to the higher nutritional content of horse blood compared to sheep blood particularly for more fastidious strain. Although excluding the COH-prepared samples did not result in statistically significant differences, the limited number of COS-treated replicates restricts the robustness of this observation. This comparison between COH and COS could not be done for HM due to the numbers of samples. This underscores the importance of standardized media and procedures in freeze-drying studies to ensure reproducibility and consistency.

Besides COH/COS media, LBS medium also presented challenges. It provided few actionable results due to suspected batch issues and dilution errors, especially given *Lactobacillus*’s subdominant presence (Heeney et al., 2018).

Donor-related factors also represent a limitation. While T5 and IG5 were tested using samples from two healthy individuals, HM was tested using stool from only one donor. Although we did not observe substantial donor-specific variability in T5 or IG5, the limited sample size restricts our ability to assess inter-individual variability in protectant performance.

Futhermore, as this was an *in vitro* and non-clinical study, infectious disease screening was not part of the experimental design. However, all donor material came from individuals previously screened and approved for clinical use, and all handling was conducted under aseptic conditions to minimize contamination risk.

Finally, viability data for stool samples without any cryo- and lyoprotectant or additional product were not collected, as no unprotected control was performed in this study. Including such data would have established a clear baseline for bacterial viability loss during sample processing and better illustrated the protective effects of each protectant formulation. Future work should incorporate unprotected controls to strengthen the comparative evaluation.

Special attention was given to bacteria of therapeutic interest, such as *Bifidobacterium*, a well-known probiotic (Wang et al., 2024). However, its exact role in treating CDI remains unclear. Restoration of gut homeostasis and control of dysbiosis likely depends more heavily on anaerobes (Baktash et al., 2018; van Nood et al., 2013). Thus, optimizing protectants for anaerobic bacteria should remain the central objective.

While this study focused primarily on bacterial viability and did not include assessments of microbial diversity via 16S rRNA sequencing or functional activity through metabolomics. Previous work (Reygner et al., 2020) has shown that freeze-drying with maltodextrin-trehalose can preserve both microbial diversity and short-chain fatty acid production, suggesting that our approach may also retain broader ecological function—though this remains to be confirmed in our setting.

Despite the limited sample size—especially for flow cytometry and HM-treated groups—these findings support further development of lyophilized bacterial therapeutics. Before clinical application, the long-term stability of freeze-dried preparations must be established before implementation. A 12-month stability study evaluating T5 at different storage temperatures (+4°C, −20°C, and −80°C) is currently underway. Preliminary results at 6 months are encouraging and will be reported in a forthcoming publication. Future research should also explore clinical efficacy after extended storage.

Taken together, these limitations highlight the need for larger, multi-center studies employing comprehensive methodologies—including viability assessments, microbial diversity analyses, and clinical outcomes—to fully optimize and validate protectant strategies for fecal microbiota transplantation.

## 5 Conclusion

Finding an ideal protectant for the freeze-drying of stool remains particularly challenging, as this complex biological matrix requires maintaining bacterial viability—a condition that is difficult to achieve. Combining flow cytometry with culture-based methods suggested viability losses during early sample processing, highlighting the importance of upstream steps in preserving microbial integrity. Our results underscore the difficulty in optimizing such formulations, especially given that the protective mechanisms of protectants are not fully understood, often leading to unpredictable effectiveness. Among the tested protectants, trehalose 5%, used in most recent studies, has consistently showed good results while HM showed potential but did not outperform T5. Regarding IG5, adding trehalose or sucrose could enhance its efficacy by providing dual protection, strengthening both extracellular and intracellular stability, thus improving bacterial survival. Ultimately, the freeze-drying of fecal microbiota preparations constitutes a pivotal step in the development of a standardized and patient-friendly oral formulation for FMT. Beyond its practical and aesthetic advantages, this strategy supports the harmonization of preparation protocols within routine clinical settings. In parallel, the advancement of next-generation fecal transplants will undoubtedly rely on comprehensive investigations into microbiota engraftment and functional outcomes, which remain critical to informing future therapeutic strategies.

## Data Availability

The raw data supporting the conclusions of this article will be made available by the authors without undue reservation.

## References

[ref1] Bai S. (2024). Enhancing the stability of probiotics: Freeze-drying and encapsulation (Doctoral Thesis (compilation), Division of Food and Pharma). Lund: Department of Process and Life Science Engineering, Lund University.

[ref2] Baktash A. Terveer E. M. Zwittink R. D. Hornung B. V. H. Corver J. Kuijper E. J.. (2018). Mechanistic insights in the success of fecal microbiota transplants for the treatment of *Clostridium difficile* infections. Front. Microbiol. 9:1242. doi: 10.3389/fmicb.2018.01242, PMID: 29946308 PMC6005852

[ref3] Bellali S. Bou Khalil J. Fontanini A. Raoult D. Lagier J.-C. (2020). A new protectant medium preserving bacterial viability after freeze drying. Microbiol. Res. 236:126454. doi: 10.1016/j.micres.2020.126454, PMID: 32200250

[ref4] Berney M. Hammes F. Bosshard F. Weilenmann H.-U. Egli T. (2007). Assessment and interpretation of bacterial viability by using the LIVE/DEAD BacLight kit in combination with flow cytometry. Appl. Environ. Microbiol. 73, 3283–3290. doi: 10.1128/AEM.02750-06, PMID: 17384309 PMC1907116

[ref5] Bilinski J. Dziurzynski M. Grzesiowski P. Podsiadly E. Stelmaszczyk-Emmel A. Dzieciatkowski T.. (2020). Multimodal approach to assessment of fecal microbiota donors based on three complementary methods. J. Clin. Med. 9:2036. doi: 10.3390/jcm9072036, PMID: 32610522 PMC7409046

[ref6] Bircher L. Geirnaert A. Hammes F. Lacroix C. Schwab C. (2018). Effect of cryopreservation and lyophilization on viability and growth of strict anaerobic human gut microbes. Microb. Biotechnol. 11, 721–733. doi: 10.1111/1751-7915.13265, PMID: 29663668 PMC6011992

[ref7] Cabeen M. T. Jacobs-Wagner C. (2005). Bacterial cell shape. Nat. Rev. Microbiol. 3, 601–610. doi: 10.1038/nrmicro1205, PMID: 16012516

[ref8] Crowe L. M. Reid D. S. Crowe J. H. (1996). Is trehalose special for preserving dry biomaterials? Biophys. J. 71, 2087–2093. doi: 10.1016/S0006-3495(96)79407-9, PMID: 8889183 PMC1233675

[ref9] Eckburg P. B. Bik E. M. Bernstein C. N. Purdom E. Dethlefsen L. Sargent M.. (2005). Diversity of the human intestinal microbial flora. Science 308, 1635–1638. doi: 10.1126/science.1110591, PMID: 15831718 PMC1395357

[ref10] Fernández-Rojas B. Gómez-Sierra T. Medina-Campos O. N. Hernández-Juárez J. Hernández-Cruz P. A. Gallegos-Velasco I. B.. (2023). Antioxidant activity of glucosamine and its effects on ROS production, Nrf2, and O-GlcNAc expression in HMEC-1 cells. Curr Res Toxicol 5:100128. doi: 10.1016/j.crtox.2023.100128, PMID: 37808439 PMC10558709

[ref11] Hamilton M. J. Weingarden A. R. Sadowsky M. J. Khoruts A. (2012). Standardized frozen preparation for transplantation of fecal microbiota for recurrent*clostridium difficile* infection. Am. J. Gastroenterol. 107, 761–767. doi: 10.1038/ajg.2011.482, PMID: 22290405

[ref12] Heeney D. D. Gareau M. G. Marco M. L. (2018). Intestinal Lactobacillus in health and disease, a driver or just along for the ride? Curr. Opin. Biotechnol. 49, 140–147. doi: 10.1016/j.copbio.2017.08.004, PMID: 28866243 PMC5808898

[ref13] Heise J. Nega M. Alawi M. Wagner D. (2016). Propidium monoazide treatment to distinguish between live and dead methanogens in pure cultures and environmental samples. J. Microbiol. Methods 121, 11–23. doi: 10.1016/j.mimet.2015.12.002 26656002

[ref14] Herdeiro R. S. Pereira M. D. Panek A. D. Eleutherio E. C. A. (2006). Trehalose protects *Saccharomyces cerevisiae* from lipid peroxidation during oxidative stress. Biochim. Biophys. Acta 1760, 340–346. doi: 10.1016/j.bbagen.2006.01.010, PMID: 16510250

[ref15] Huang L. Lu Z. Yuan Y. Lü F. Bie X. (2006). Optimization of a protective medium for enhancing the viability of freeze-dried *Lactobacillus delbrueckii* subsp. bulgaricus based on response surface methodology. J. Ind. Microbiol. Biotechnol. 33, 55–61. doi: 10.1007/s10295-005-0041-8, PMID: 16244855

[ref16] Hubálek Z. (2003). Protectants used in the cryopreservation of microorganisms. Cryobiology 46, 205–229. doi: 10.1016/s0011-2240(03)00046-4 12818211

[ref17] International Organization for Standardization (2024). ISO 7218:2024: microbiology of food and animal feeding stuffs — General requirements and guidance for microbiological examinations. Available at: https://www.iso.org/standard/79508.html

[ref18] Jofré A. Aymerich T. Garriga M. (2015). Impact of different cryoprotectants on the survival of freeze-dried Lactobacillus rhamnosus and *Lactobacillus casei*/paracasei during long-term storage. Benef Microbes 6, 381–386. doi: 10.3920/BM2014.0038, PMID: 25380798

[ref19] Kapel N. Waligora-Dupriet A. -J. Thomas M. Charrueau C. Joly F. Mayeur C.. (2017) Lyophilized composition for preserving microbiota in its ecosystem. WO/2017/103225. Available at: https://patentscope.wipo.int/search/en/WO2017103225

[ref20] Kelly C. R. Khoruts A. Staley C. Sadowsky M. J. Abd M. Alani M.. (2016). Effect of fecal microbiota transplantation on recurrence in multiply recurrent *Clostridium difficile* infection. Ann. Intern. Med. 165, 609–616. doi: 10.7326/M16-0271, PMID: 27547925 PMC5909820

[ref21] Lee C. H. Steiner T. Petrof E. O. Smieja M. Roscoe D. Nematallah A.. (2016). Frozen vs fresh fecal microbiota transplantation and clinical resolution of diarrhea in patients with recurrent *Clostridium difficile* infection: a randomized clinical trial. JAMA 315, 142–149. doi: 10.1001/jama.2015.18098, PMID: 26757463

[ref22] Miyamoto-Shinohara Y. Sukenobe J. Imaizumi T. Nakahara T. (2008). Survival of freeze-dried bacteria. J. Gen. Appl. Microbiol. 54, 9–24. doi: 10.2323/jgam.54.9 18323678

[ref23] Nichols K. Holmes A. (2007). “CHAPTER 21 - non-parametric procedures” in Statistical parametric mapping. eds. Friston K. Ashburner J. Kiebel S. Nichols T. Penny W. (London: Academic Press), 253–272.

[ref24] Oluwatosin S. O. Tai S. L. Fagan-Endres M. A. (2021). Sucrose, maltodextrin and inulin efficacy as cryoprotectant, preservative and prebiotic – towards a freeze dried *Lactobacillus plantarum* topical probiotic. Biotechnol. Rep. (Amst.) 33:e00696. doi: 10.1016/j.btre.2021.e00696 35024350 PMC8732778

[ref25] Qin Y.-Q. Wang L.-Y. Yang X.-Y. Xu Y.-J. Fan G. Fan Y.-G.. (2023). Inulin: properties and health benefits. Food Funct. 14, 2948–2968. doi: 10.1039/D2FO01096H 36876591

[ref26] Reygner J. Charrueau C. Delannoy J. Mayeur C. Robert V. Cuinat C.. (2020). Freeze-dried fecal samples are biologically active after long-lasting storage and suited to fecal microbiota transplantation in a preclinical murine model of *Clostridioides difficile* infection. Gut Microbes 11, 1405–1422. doi: 10.1080/19490976.2020.1759489, PMID: 32501140 PMC7524285

[ref27] Rockinger U. Funk M. Winter G. (2021). Current approaches of preservation of cells during (freeze-) drying. J. Pharm. Sci. 110, 2873–2893. doi: 10.1016/j.xphs.2021.04.018, PMID: 33933434

[ref28] Rodríguez Furlán L. T. Lecot J. Pérez Padilla A. Campderrós M. E. Zaritzky N. (2011). Effect of saccharides on glass transition temperatures of frozen and freeze dried bovine plasma protein. J. Food Eng. 106, 74–79. doi: 10.1016/j.jfoodeng.2011.04.010

[ref29] Staley C. Hamilton M. J. Vaughn B. P. Graiziger C. T. Newman K. M. Kabage A. J.. (2017). Successful resolution of recurrent *Clostridium difficile* infection using freeze-dried, encapsulated fecal microbiota; pragmatic cohort study. Am. J. Gastroenterol. 112, 940–947. doi: 10.1038/ajg.2017.6, PMID: 28195180 PMC5552199

[ref30] Stefanello R. F. Machado A. A. R. Pasqualin Cavalheiro C. Bartholomei Santos M. L. Nabeshima E. H. Copetti M. V.. (2018). Trehalose as a cryoprotectant in freeze-dried wheat sourdough production. LWT 89, 510–517. doi: 10.1016/j.lwt.2017.11.011

[ref31] Stefanello R. F. Nabeshima E. H. Iamanaka B. T. Ludwig A. Fries L. L. M. Bernardi A. O.. (2019). Survival and stability of Lactobacillus fermentum and Wickerhamomyces anomalus strains upon lyophilisation with different cryoprotectant agents. Food Res. Int. 115, 90–94. doi: 10.1016/j.foodres.2018.07.044, PMID: 30599986

[ref32] Stiefel P. Schmidt-Emrich S. Maniura-Weber K. Ren Q. (2015). Critical aspects of using bacterial cell viability assays with the fluorophores SYTO9 and propidium iodide. BMC Microbiol. 15:36. doi: 10.1186/s12866-015-0376-x, PMID: 25881030 PMC4337318

[ref33] Sträuber H. Müller S. (2010). Viability states of bacteria--specific mechanisms of selected probes. Cytometry A 77, 623–634. doi: 10.1002/cyto.a.20920 20583280

[ref34] Suau A. Bonnet R. Sutren M. Godon J. J. Gibson G. R. Collins M. D.. (1999). Direct analysis of genes encoding 16S rRNA from complex communities reveals many novel molecular species within the human gut. Appl. Environ. Microbiol. 65, 4799–4807. doi: 10.1128/AEM.65.11.4799-4807.1999, PMID: 10543789 PMC91647

[ref35] Tawfick M. M. Xie H. Zhao C. Shao P. Farag M. A. (2022). Inulin fructans in diet: role in gut homeostasis, immunity, health outcomes and potential therapeutics. Int. J. Biol. Macromol. 208, 948–961. doi: 10.1016/j.ijbiomac.2022.03.218, PMID: 35381290

[ref36] van Nood E. Vrieze A. Nieuwdorp M. Fuentes S. Zoetendal E. G. de Vos W. M.. (2013). Duodenal infusion of donor feces for recurrent *Clostridium difficile*. N. Engl. J. Med. 368, 407–415. doi: 10.1056/NEJMoa1205037, PMID: 23323867

[ref37] Wang Y. Hunt A. Danziger L. Drwiega E. N. (2024). A comparison of currently available and investigational fecal microbiota transplant products for recurrent *Clostridioides difficile* infection. Antibiotics (Basel) 13:436. doi: 10.3390/antibiotics13050436, PMID: 38786164 PMC11117328

[ref38] Wang G. Luo L. Dong C. Zheng X. Guo B. Xia Y.. (2021). Polysaccharides can improve the survival of Lactiplantibacillus plantarum subjected to freeze-drying. J. Dairy Sci. 104, 2606–2614. doi: 10.3168/jds.2020-19110, PMID: 33309373

[ref39] Xing R. Liu S. Guo Z. Yu H. Li C. Ji X.. (2006). The antioxidant activity of glucosamine hydrochloride in vitro. Bioorg. Med. Chem. 14, 1706–1709. doi: 10.1016/j.bmc.2005.10.018, PMID: 16263299

[ref40] Youngster I. Russell G. H. Pindar C. Ziv-Baran T. Sauk J. Hohmann E. L. (2014). Oral, capsulized, frozen fecal microbiota transplantation for relapsing *Clostridium difficile* infection. JAMA 312, 1772–1778. doi: 10.1001/jama.2014.13875, PMID: 25322359

[ref41] Zain N. M. M. ter Linden D. Lilley A. K. Royall P. G. Tsoka S. Bruce K. D.. (2022). Design and manufacture of a lyophilised faecal microbiota capsule formulation to GMP standards. J. Control. Release 350, 324–331. doi: 10.1016/j.jconrel.2022.08.012, PMID: 35963468

[ref42] Zhang T. Fang H. H. P. (2004). Quantification of *Saccharomyces cerevisiae* viability using BacLight. Biotechnol. Lett. 26, 989–992. doi: 10.1023/b:bile.0000030045.16713.19, PMID: 15269525

