# Supplementary figures and images for: Standardized freeze-dried FMT: is the ideal protectant out there?
Source: Front Microbiol. 2025 Aug 13;16:1618067. doi: 10.3389/fmicb.2025.1618067 (PMC12380771; doi:10.3389/fmicb.2025.1618067)

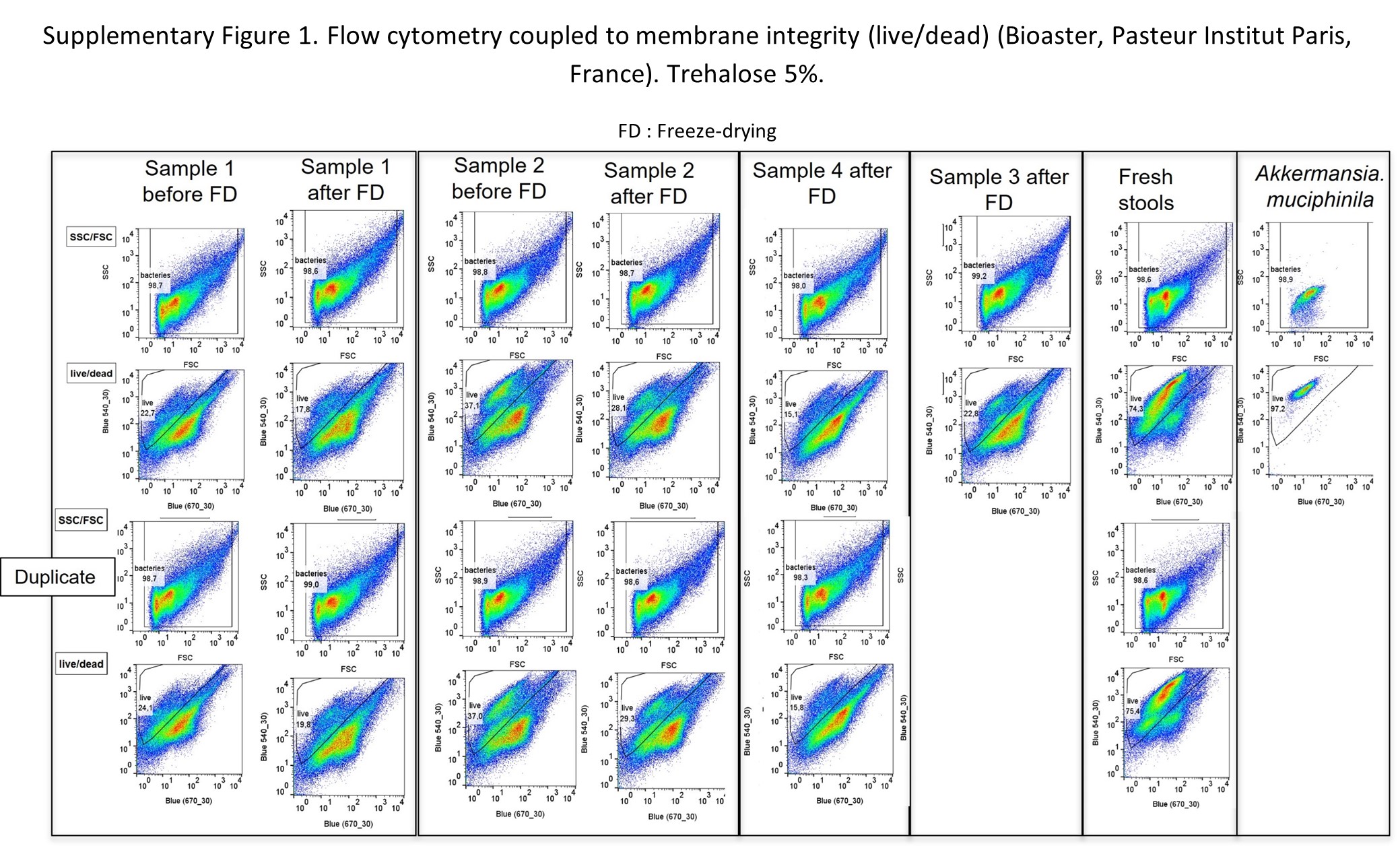

Supplement: Supplementary file 1 [file Image_1.jpeg]
