# Supplementary material for: Standardized freeze-dried FMT: is the ideal protectant out there?
Source: Front Microbiol. 2025 Aug 13;16:1618067. doi: 10.3389/fmicb.2025.1618067 (PMC12380771; doi:10.3389/fmicb.2025.1618067)
Supplement: Supplementary file 2 [file Table_1.docx]

Supplementary table 1. Microorganism (MO) enumeration by flow cytometry analysis (FD = Freeze-drying). Data are mean of duplicates. Akkermansia was used as a control.

| **Samples** | **MO/g of stool** | **MO/g of pre-lyophilized stools** | **MO/g of lyophilized stools** | **Viability (%)** |
| --- | --- | --- | --- | --- |
| Sample 1 (before ED) | - | 3.51 x10^10^ | - | 23,4% |
| Sample 1 (after ED) | - | - | 3.77 x10^11^ | 18,8% |
| Sample 2 (before ED) | - | 5.33 x10^10^ | - | 37,1% |
| Sample 2 (after ED) | - | - | 5.36 x10^11^ | 28,7% |
| Sample 3 (after FD) | - | - | 4.00 x10^11^ | 21,3% |
| Sample 4 (after FD) | - | - | 3.03 x10^11^ | 15,5% |
| Fresh Stool | 9.07 x10^11^ | - | - | 74,9% |

Supplementary table 2. Bacterial viability by plating (Colony Forming Unit CFU) on Tryptone Soy Agar (TSA) for total aerobe bacteria and on Drigalski (DRI) for *Enterobacteria*, before freeze-drying in the total stool pellet and after freeze-drying in the total lyophilized stool obtained with the HM (High-antioxidant Matrix: Trehalose- ascorbic acid-sorbitol-glutamine) cryoprotectant.

|  | **Agar** | **Bacterial count before freeze-dry in pellet (CFU)** | **Bacterial count after freeze-dry in lyophilized stool (CFU)** |
| --- | --- | --- | --- |
| Sample HM1 | TSA | 5,60E+11 | 4,40E+11 |
| Sample HM2 | TSA | 2,11E+10 | 4,70E+10 |
| Sample HM3 | TSA | 4,06E+10 | 3,80E+10 |
| Sample HM4 | TSA | 2,11E+10 | 4,42E+10 |
| Sample HM1 | DRI | 3,85E+11 | 3,26E+11 |
| Sample HM2 | DRI | 3,11E+10 | 3,53E+10 |
| Sample HM3 | DRI | 3,10E+10 | 2,27E+10 |
| Sample HM4 | DRI | 1,76E+10 | 3,44E+10 |
